# Plant use and perceptions in the context of sexual health among people of Congolese descent in Belgium

**DOI:** 10.1186/s13002-024-00662-3

**Published:** 2024-02-19

**Authors:** Laura Van Damme, Lars Chatrou, Eduardo de la Peña, Pathy Kibungu, Césarine Sinatu Bolya, Patrick Van Damme, Wouter Vanhove, Melissa Ceuterick, Emiel De Meyer

**Affiliations:** 1 Department of Biology, Ghent University, K.L. Ledeganckstraat 35, 9000 Ghent, Belgium; 2 Department of Plants and Crops, Ghent University, Coupure Links 653, 9000 Ghent, Belgium; 3 Spanish National Research Council (CSIC), Institute for Subtropical and Mediterranean Horticultural Research (IHSM-UMA-CSIC), Malaga, Spain; 4 Natural Products & Food Research and Analysis (NatuRA), Department of Pharmaceutical Sciences, University of Antwerp, Universiteitsplein 1, 2610 Antwerp, Belgium; 5 Department of Environmental Sciences, Kinshasa University (UNIKIN), BP 127, Kinshasa XI, Democratic Republic of the Congo; 6 Brussels, Belgium; 7 Faculty of Tropical AgriSciences (FTA), Czech University of Life Sciences Prague, 165 00 Prague, Czech Republic; 8 Lignaverda Belgium, Duwijckstraat 17, 2500 Lier, Belgium; 9 Department of Sociology, Health & Demographic Research, Ghent University, Sint-Pietersnieuwstraat 41, 9000 Ghent, Belgium

**Keywords:** Ethnobotany, Medicinal plants, Democratic Republic of Congo, Sexual health

## Abstract

**Background:**

The use of medicinal plants is integral to global healthcare systems, with Sub-Saharan Africa maintaining a robust tradition of herbal medicine alongside Western-oriented healthcare. As migrant communities tend to continue traditional herbal practices after migration, documenting this use is vital to develop culturally sensitive healthcare. This study investigates plant usage and perspectives in the context of sexual and reproductive health among the Congolese community in Belgium, particularly in the Matongé quarter of Brussels. Our research questions were: (1) What is the current knowledge of medicinal plants among the Congolese community in Belgium in the context of sexual health, and what are the applications and commonly employed administration methods of these plants? (2) What role does herbal medicine play in the context of sexual health for people of Congolese descent in Belgium and how this is influenced by perceptions of sexuality? and (3) Is there a gender bias in the use of medicinal plants, and if so, can this be related to perceived gender norms?

**Methods:**

We conducted 22 semi-structured interviews with people of Congolese descent currently living in Belgium. Participants were selected using both snowball sampling and purposive sampling. Plant use in the context of sexual health was recorded through freelisting. Data on narratives, ideas, and perceptions of this plant use in the context of sexual health were collected. Interview transcripts were analyzed using thematic analysis.

**Results:**

We identified 17 plant species used for sexual health. Three overarching themes emerged from our data. Plants were used with a notable gender bias favoring male sexual potency enhancement. Men used these plants for both remedying potency issues and enhancing sexual prowess. In contrast, knowledge about plants for female sexual health was limited. Gender norms reinforced the importance of male sexual potency, while stigmatizing open discussions of female sexuality.

**Conclusions:**

The use of medicinal plants for sexual health raises health, social, and conservation concerns, underscoring the need for further research in this area. This study contributes to understanding medicinal plant use within the Congolese community in Belgium and highlights the necessity for future research on herbal practices for female sexual health in this context.

## Background

Since time immemorial, people have relied on plants for medicinal purposes [1] and to this day, herbal medicine functions as an indispensable element of the global healthcare system [1, 2]. The use of medicinal plants is not limited to regions with restricted access to allopathic medicine. Several studies have documented that in urban Sub-Saharan Africa, herbal medicine usage remains prevalent, even when Western-based healthcare facilities are available [3–6]. The latter trend is also visible in Western urban settings, where herbal medicine and other types of traditional, complementary, and alternative medicine have even been growing in popularity [7–9]. This globally increasing popularity has resulted in a growing demand of herbal medicine, and has made the trade in medicinal plants a booming sector over the past decades [10, 11].

The global trade in herbal products is partially driven by increasing international (and national) migration. Research among ethnic minorities in Europa and the USA has demonstrated that migrants tend to at least partly maintain their traditional practices and perceptions regarding health and healthcare after migration [5, 12, 13]. The continued use of medicinal plants can be seen as a way to remain in touch with one’s cultural background in the host country, but can also be motivated by health considerations, when medicinal plants are believed to be more effective than other types of medication, or can be driven by a perceived lack of alternative acceptable allopathic healthcare options [14]. Documenting the continued use of traditional herbal medicine offers valuable insights into how individuals with a migration background integrate their traditional health practices within the prevailing healthcare landscape of their current geographical context. This holds particular significance as health disparities persist among different ethnicities in Europe. While there is no uniform trend across Europe, several countries, including Belgium, have documented poorer health status and reduced access to preventative healthcare services among immigrants, despite the presence of universal health care [15, 16]. A particularly significant disparity is evident in the prevalence of sexually transmitted diseases, specifically HIV. Individuals of Sub-Saharan African descent living in Belgium are disproportionately affected, constituting the second largest group within newly reported HIV cases in 2019 [17].

In Belgium, the Congolese community forms the largest community of Sub-Saharan African descent, with an estimated 90,000 people of Congolese descent living in Belgium in 2022 (Figures from the National Register, drawn up by Statbel, edited and calculated by Myria; personal communication, 2022). This migration is intrinsically linked to the shared colonial history between Belgium and the Democratic Republic of the Congo (henceforth DR Congo) [18, 19]. After independence in 1960, Congolese students and expats have mainly resided in an area that is now referred to as the Matongé quarter in Ixelles, Brussels [20]. This predominantly Congolese neighborhood is now known as a vibrant, multicultural meeting place, as well as a commercial hub [21]. The few streets and galleries harbor plenty of hair salons, African restaurants and bars, shops selling clothes and cloth, wigs and hair extensions, as well as alimentation shops run by people from varying nationalities [22]. Besides, the Matongé quarter has been identified as a hub for both formal and informal trade between Central Africa and Europe [23]. In this context, medicinal plant trade in Matongé was documented by Van Andel and Fundiko [24]. However, there still remains a significant knowledge gap in the use of these plants within the Congolese community in Belgium [24, 26].

Recent informal news sources have highlighted the rising use of libido-enhancing plants in Matongé [27], a trend supported by Van Andel and Fundiko's research [24] on African medicinal plant applications. Ethnobotanical studies across Sub-Saharan Africa, including DR Congo, have consistently documented plant use for various sexual health purposes, such as aphrodisiacs, vaginal cleanses, fertility enhancement, postpartum recovery, and sexual transmitted disease treatment [28–31]. Simultaneously, various news outlets have highlighted the increasing trade of sexually stimulating plants in Kinshasa [32–34]. However hitherto, no study has directly addressed medicinal plant use in a sexual health context among people of Congolese descent in Belgium. Therefore, the main objective of this study was to gain insight in the plant use and perspectives in the context of sexual and reproductive health among the Congolese community in Belgium.

This led us to the following research questions:

1. What is the current knowledge of medicinal plants among the Congolese community in Belgium in the context of sexual health, and what are the applications and commonly employed administration methods of these plants?
2. What role does herbal medicine play in the context of sexual health for people of Congolese descent in Belgium and how this is influenced by perceptions of sexuality?
3. Is there a gender bias in the use of medicinal plants, and if so, can this be related to perceived gender norms?

## Methods

### Data collection

Data collection took place between September 2022 and January 2023. Semi-structured in-depth interviews were conducted with people of Congolese descent in Belgium. Eligibility criteria were: a minimum age of 18 years, first- or second-generation Congolese (DR Congo) immigrant, and residing primarily in Belgium. The first set of participants was formed by approaching individuals, mainly in the Matongé quarter, Brussels. This quarter was chosen as main participant-recruiting location since previous research highlighted Matongé as a focal for Congolese medicinal plant use and knowledge [24, 26]. Starting from this first set of participants, other participants were recruited through snowball sampling [35], based on which we performed further purposive sampling. Snowball sampling was initiated from different starting points in order to avoid sampling within a restricted network. A purposive sample was designed to obtain maximum variation in gender, age, and professional background [36]. Eventually, participants were selected from the lists of potential interviewees (acquired by snowball sampling) based on sampling criteria that were still missing. In addition to this, people who mainly claimed to have knowledge about medicinal plants or perceptions about links between plant use and sexuality were also selected. The sampling process was considered complete when conducting three additional interviews failed to reveal any new themes, indicating data saturation [37]. Most interviews took place in person. Three interviews were conducted online using videotelephony software. After obtaining oral informed consent, all interviews were audio-recorded. After every interview, participants were additionally requested to sign an informed consent document. Interviews were subsequently fully transcribed and anonymized. Interview questions were developed consulting two key informants, tested in two pilot interviews, and subsequently finalized. During the interviews, participants were asked to free-list medicinal plants they know and/or use for disease prevention or treatment related to sexual and reproductive health. Only plants of which the use in Belgium was confirmed by participant testimonials and/or through proven availability of plant material, were included in the dataset. Used plant parts and preparation methods were also noted. Besides, questions were asked about why participants used specific medicinal plants and about participants’ perceptions of sexuality, gender norms, and their link with plants used in the context of sexual health.

### Data analysis

Interview transcripts were analyzed in the language in which they were conducted. Qualitative data were analyzed using thematic analysis [38]. Given the exploratory nature of this study, we deemed this approach particularly relevant to identify prevailing ideas and practices. Transcripts were coded in different phases as described by Clarke and Braun [38]. Draft themes were discussed with three authors in an iterative process and led to three final themes, which are elaborated in the Results section. Relevant parts of the transcripts were translated into English for their use as quotes.

In order to help overcome some of the limitations of the cultural outsider position of the interviewer, we consulted two key informants from the Congolese community throughout the research process, which aided us to deepen our understanding of the cultural context and mitigate biases in research design, data collection, and data analysis.

### Plant identification

All mentioned plants were searched in shops and through street vendors in Matongé and where possible collected into a voucher collection of dried plant specimens. Physical plant samples were either offered by participants or purchased from vendors of African alimentation shops or informal traders. Collected plant samples were identified using the PROTA4U database [39], in combination with The Flora of Central Africa [40]. Plant species mentioned that were not physically collected during the interview period, as well as plant species for which only vernacular names (in French or Lingala) were provided but whose connection with collected plant samples was unclear, were identified through positive visual identification. The latter was done by searching for clearly identified pictures of the plant habitus and plant parts with participants to confirm plant identity and their correct scientific name. The accuracy of the listed vernacular names was later checked during an ethnobotanical literature search on Congolese medicinal plants. Plant scientific names were checked for using the IPNI database [41].

### Literature review

The list of identified plant species and their families was compared with ethnobotanical literature on Congolese medicinal plant use. An exhaustive online literature search was conducted using a comprehensive approach that covered both peer-reviewed scientific articles and gray literature. The search involved keywords such as *DR Congo*, *ethnobotany*, *medicinal plants*, *sexual dysfunction*, *aphrodisiac,* and *impotence*, in both English and French. Databases such as Web of Science, Google Scholar, and PubMed were consulted to retrieve a wide range of relevant information. The literature search encompassed general research articles on medicinal plants in DR Congo, as well as more specific studies on the use of herbal medicine in particular regions or for specific applications.

## Results

### Interviews

Twenty-two participants, consisting of 14 men and 8 women (Table 1), were interviewed, with interview durations ranging from 14 min to 3 h (median: 28.5 min). Interviews were conducted in French or Dutch based on participant preferences. The interview duration varied depending on participants' plant knowledge, experiences, and their willingness to discuss factors influencing medicinal plant use. Nineteen participants were recruited in or through contacts located in the Matongé quarter, three were recruited outside the quarter. The majority (17) were first-generation Congolese migrants, while five were second-generation migrants, born in Belgium with at least one parent from DR Congo. Respondent ages ranged from 22 to 74 years, with women having a higher average age (56) compared to men (38).

**Table 1:** Informant profiles, SD: standard deviation

Factors	Male	Female
Number of participants	14	8
Age range (years)	26–67	22–74
Median age (years)	38 (SD: 14.6)	56 (SD: 15.7)
First generation in Belgium	10	7
Second generation in Belgium	4	1
Total number of participants	22	

At the time of the interviews, six participants worked in or owned a local business in Matongé, including hair salons, clothing stores, or small grocery stores. Four participants were retired, three were students, and three were unemployed. Remaining participants performed various jobs in the tertiary sector. Seventeen out of 22 of participants lived in and around the city of Brussels. The others lived in or near the Flemish cities of Ostend, Antwerp, and Ghent. Informant profiles are presented in Table 1.

### Taxonomic diversity

We inventoried seventeen different plant species and plant products, with 14 identified to the species level and one to the genus level. Plant species were classified in 12 families. Two herbal products, locally named “*ankoro*” and “*mogomboro*,” could not be identified. Families Malvaceae (*Cola acuminata* (P.Beauv.) Schott and Endl*., Abelmoschus esculentus* (L.) Moench*, Theobroma cacao* L.), and Zingiberaceae (*Zingiber officinale* Roscoe*, Aframomum melegueta* K.Schum.) were the most represented, by three and two species, respectively. Most of the plant material was sold in a dry form, except for the species primarily sold for culinary uses, including *Z. officinale*, *Cucumis sativus* L. and *A. esculentus*. A list of all inventoried plant species, along with their respective families, genera, vernacular names, uses, preparation and administration methods, number of records, and references to their documented use in Congolese ethnobotanical studies, is presented in Table 2. Moreover, participants reported the utilization of the processed herbal product “*Asili Power Plus—haute énergie*,” a commercial drink imported from Kinshasa that contains *Z. officinale* and *Eurycoma longifolia* Jack ('Tongkat Ali'), renowned for its aphrodisiac properties [42]. One woman mentioned using (Potassium) permanganate (KMnO4) for vaginal infections, while Viagra® was mentioned by two participants in the context of sexuality. Non-herbal products, along with their applications, methods of application, and the number of mentions, are detailed in Table 2. Publications confirming the use of each plant species for medicinal purposes in DR Congo as well as a specification of whether this use encompasses sexual health applications are also included in Table 2.

**Table 2:** List of products used in the context of sexual health by the Congolese community in Belgium

	Family	Species	Vernacular name	Main application	M/F	Plant part	Mode of preparation	Added species	Number of mentions	Cited in Congolese literature	Sexual health applications in Congolese literature
1	Amaryllidaceae	*Allium sativum* L	ail (Fr.)	Erectile dysfunction	M	Bulb	Direct ingestion	*Z. officinale, M. whitei, H. crinita*	2	[3, 51, 31, 49, 78]	Impotence (8)
2	Anacardiaceae	*Mangifera indica* L	Mango (Fr.)	Vaginal infection	F	Leaf	Vaginal suppository	*P. guineense*	2	[51, 31, 76, 78, 54]	Impotence (11) Vaginal infection (13) Tightening vagina (13)
3	Apocynaceae	*Mondia whitei* (Hook.f.) Skeels	Kimbiologo (Li.)	Libido enhancement	M	Root	Infusion, direct ingestion	*Z. officinale*	12	[3, 51, 31, 49, 78, 50]	Impotence (1,8,12,14) UTI (12) Not specified (4)
				Erectile dysfunction	M						
4	Clusiaceae	*Garcinia kola* Heckel	Ngadiadia (Li.), Petit Cola (Fr.)	Libido enhancement	M	Seed	Direct ingestion		6	[3, 51, 75, 49, 78, 50]	Impotence (5,8,14)
5	Cucurbitaceae	*Cucumis sativus* L	Concombre (Fr.)	Libido enhancement	M	Fruit	Direct ingestion		2	Not documented	–
6	Lamiaceae	*Ocimum gratissimum* L	Lumba Lumba (Li.)	Vaginal infection	F	Leaf	Vaginal suppository		1	[3, 31, 49, 78, 54]	Vaginal infection (13) Tightening vagina (13)
7	Malvaceae	*Cola acuminata* (P.Beauv.) Schott & Endl	Makasu (Li.), Noix de Cola (Fr.)	Libido enhancement	M	Seed	Direct ingestion		8	[51, 31, 49, 77, 78, 50]	Impotence (10,12,14) Not specified (4)
8		*Abelmoschus esculentus* (L.) Moench	Dongodongo (Li.)	Libido enhancement	M	Fruit	Direct ingestion		3	[3, 76, 49, 78]	–
				Smooth delivery	F				1		
9		*Theobroma cacao* L	Cacao (Fr.)	Libido enhancement	M	Seed	Direct ingestion		1	Not documented	–
10	Piperaceae	*Piper guineense* Schumach. & Thonn	Ketschu (Li.), Poivre sauvage (Fr.)	Vaginal infection	F	Fruit	Vaginal suppository	*M. indica*	1	[49, 77, 78, 50]	Impotence (14)
11	Poaceae	*Cymbopogon citratus* (DC.) Stapf	Sinda (Li.)	Libido enhancement	M	Leaf	Infusion		1	[3, 51, 31, 75, 49, 78]	–
12	Rubiaceae	*Heinsia crinita* (Wennberg) G. Taylor	Kita Mata (Li.)	Erectile dysfunction	M	Root	Infusion, direct ingestion	*Z. officinale*	11	[3, 51, 75, 49, 78, 54, 50]	Impotence (1,2,8,11,14) Female infertility (5) Vaginal infection (13) Tightening vagina (13)
				Premature ejaculation	M						
13	Solanaceae	*Capsicum* spp.	Piment (Fr.)	Libido enhancement	M	Fruit	Direct ingestion		1	[3, 51, 31, 75, 49, 77, 78]	–
				Vaginal infection	F		Vaginal suppository		1		
14	Zingiberaceae	*Zingiber officinale* Roscoe	Tangawisi (Li.)	Libido enhancement	M	Rhizome	Infusion, direct ingestion	*A. sativum, M. whitei, H. crinita*	18	[3, 51, 31, 49, 54, 50]	Impotence (2,8,11,14) Vaginal infection (13) Tightening vagina (13) Not specified (4)
				Vaginal infection	F		Vaginal suppository		2		
				Tightening vagina	F		Vaginal suppository				
15		*Aframomum melegueta* K.Schum	Mondongo (Li.)	Libido enhancement	M	Seed	Direct ingestion		6	[3, 51, 31, 49, 54, 50]	Female infertility (8) Impotence (14) Libido enhancement (12) Not specified (4)
16	unidentified	unidentified	Mogomboro (Li.)	Enlarging penis	M	N.A	N.A		4	[24, 25]	–
17	unidentified	unidentified	Ankoro (Li.)	Premature ejaculation	M	Bark	Direct application		12	Not documented	–
18	N.A	N.A	Asili Power Plus haute énergie	Libido enhancement	M	N.A	Direct ingestion		1	–	
*Non-plant products*											
19	N.A	N.A	Viagra®	Erectile dysfunction	M	N.A	Direct ingestion		2		
20	N.A	N.A	Permanganate (Fr.)	Vaginal infection	F	N.A	Vaginal suppository		1		

Vernacular name: Fr.: French, Li.: Lingala; M: male, F: female; N.A: not applicable

The thematic analysis resulted in the identification of the following three overarching themes: (1) Plants, medicine or sexual enhancer? (2) Pressure to have strong sexual potency, and (3) Taboos and social norms surrounding female sexuality.

#### Theme 1: plants, medicine or sexual enhancer?

Almost all (21 out of 22) participants reported to be familiar with one or more Congolese plants used for sexual and reproductive health purposes. Only half of the participants reported having personal experience with the use of one or several of these plant species to enhance sexual health.

##### Male potency enhancers

Regarding male potency enhancers, *Z. officinale* (ginger), specifically a Congolese variety producing small rhizomes directly imported from the DR Congo, was the most frequently mentioned and used plant species (Table 2). The vast majority of participants stated that frequent consumption of *ginger* gives and helps men to maintain sexual energy. Several participants referred to ginger as the basis of herbal medicine for male sexual performance:

“They will prepare stuff with mostly ginger, that is never lacking. It’s the basis for men.” – Man, 41, first generation in Belgium

Ginger was mostly consumed in the form of a drink called ‘*Tangawisi’* and often mixed with the roots of other herbs known for boosting sexual potency such as *Mondia whitei* (Hook.f.) Skeels and *Heinsia crinita* (Wennberg) G.Taylor. These plants were more specifically mentioned to help maintain an erection and increase the semen volume. Twelve participants mentioned the herbal product *ankoro*, a numbing powder, which is directly applied on the penis to prolong erection and prevent premature ejaculation. The majority (14 out of 17) of reported plants were primarily used for male sexuality. Enhanced sexual desire (10 out of 17 species), treatment of erectile dysfunction (three species), and prevention of premature ejaculation (two species) emerged as the predominant motivations for the utilization of medicinal plants among men.

Various explanations were given as to why plants contributing to male sexuality are so widely known and frequently used or experimented with. Six participants expressed their belief that a clear distinction could be made between users of these medicinal plants. According to them, the demand is on the one hand partly driven by men who experience sexual dysfunction and are looking to restore or improve their sexual potency. On the other hand, these plants are consumed by a group of men who claim to be fully sexually performant in order to further improve and explore their sexual performance:

“There are people that use it simply because they need it and there are people that use it because they want to have fun tonight.” – Man, 28, second generation in Belgium

All second-generation Congolese men primarily considered the listed plants as remedies for sexual impotence, showing limited interest due to their belief in their inherent strong sexual potency during youth. In contrast, first-generation Congolese men, including younger individuals, had a more positive attitude toward using herbal products to enhance sexual performance. They had experience with these plants, with half of them reportedly consuming them for sexual purposes despite already having strong sexual potency, thus as a way to further enhance their usual sexual performance rather than as a remedy. Multiple participants described this difference in perception between those with Congolese roots born in Belgium and those with closer ties to DR Congo.

“For me who grew up here it's not something we discussed among friends. We didn't really have a need for these products. But when I see, for example the others, my cousins in Africa, for them it's something they discuss. For example: they will go out with a girl, if it’s not an important girl, it’s fine. But if it's an important girl who has to fall in love with them: it’s part of it. The same way as a condom, it's part of the game. It's something you will use because it makes you stronger.” – Man, 48, second generation in Belgium

##### Female remedies

Plants used by women to boost or restore sexual and reproductive health were less commonly mentioned (by only 3 out of 22 participants). Five plant species were reportedly used as vaginal suppository to combat infections. These plants included *Z. officinale, Piper guineense* Thonn. and leaves of *Mangifera indica* L. These practices were shared by three women over 60 years old, who learned them in DR Congo. They confirmed continuing these practices after moving to Belgium but noted limited access to fresh plant materials and decreased interest due to the growing availability of allopathic remedies compared to medicinal plants.

“In Congo, for example, in our youth, there was no alternative to using herbs in case of vaginal inflammation or infection. At that time, we didn't have any pharmaceutical liquid to disinfect the vagina.” – Woman, 74, first generation in Belgium

Various explanations were given for why most of the listed plants targeted male rather than female sexual health. Ten participants suggested that libido-stimulating plants were unnecessary for women. Two men argued that women experience sexual arousal naturally and with less effort, while four men believed that women, being the stronger sex, have greater sexual stamina. Consequently, certain medicinal plants, such as *ankoro* and *H. crinita*, were consumed by men to prolong sexual intercourse, aiming to match female sexual stamina. In contrast, one young woman found a possible explanation in her experience that in African cultures, there tends to be a stronger focus on satisfying male needs.

“We admit that women are stronger than us. […] A woman can last longer than us during sex. That’s why I think it’s more targeted towards men.” – Man, 26, second generation in Belgium

Four men believed that during copulation, men do most of the physical activity, making their potency a crucial factor for successful sexual intercourse. Two of them also added that difficulties related to intercourse are mainly a result of male sexual potency issues, making herbal medicines aimed at enhancing sexual performance more useful for men than for women.

“In our culture, in our conception of the matter, the sexual act is above all a man's business. Because for most of the problems related to sex, it’s simply the man that is not performant.” – Man, 37, first generation in Belgium

Three participants suggested that the lower demand for libido-stimulating plants among women may be due to the perception that female sexuality is taboo within the Congolese community. One female participant attributed the gender-biased availability of such plants to differences in how men's and women's sexual desires function. She suggested that female desire is more influenced by mental factors, potentially making plants less effective for women. However, six participants countered this idea, suggesting that libido-stimulating plants could also work for and be used by women. Nevertheless, all of them noted that women generally have less interest in using plants for sexual purposes.

### Ethnobotanical literature search

Extensive evidence from Congolese ethnobotanical literature supports the use of nearly all listed plant species for various medical purposes. Only two species, *C. sativus* and *T. cacao,* were not mentioned in the literature. The vernacular name *ankoro* was also absent from ethnobotanical literature. The vernacular name *mogomboro* is mentioned a few times in the scientific ethnobotanical literature but it is linked with multiple plant species [21, 25]. While most plants identified by participants for enhancing male sexual potency had documented uses in DR Congo, exceptions included *Cymbopogon citratus* (DC.) Stapf, *A. esculentus* (L.), and *Capsicum* spp. Approximately half of the plants associated with female sexual and reproductive health had similar documented uses in DR Congo. However, one participant believed *P. guineense* and *Capsicum* spp. were effective against vaginal infections, a property not found in the literature. Likewise, there were no reports found of *A. esculentus* aiding in smooth deliveries.

#### Theme 2: pressure to have strong sexual potency

Five men expressed the belief that people of African descent are more sexually performant than people of other than African descent. Similarly, two participants believed that people of African descent are more focused on sexuality compared to Europeans.

“You have to understand that Africans are by nature very strong sexually. It is natural. It is not because of materials like that. So, there are some who additionally take these products, and there are some who don't.” – Man, 67, first generation in Belgium

“Here in Europe, sex is not a priority, but in Africa….” – Woman, 74, first generation in Belgium

Three respondents found an explanation for this perception in the fact that numerous plants used in the traditional African cuisine are known to have libido-stimulating properties. Because of the latter, the bodies of people of African descent are thought to be prepared from a young age to become sexually performant.

“There are many vegetables in the regular African nutrition that are stimulating products. That’s why there is perhaps the myth that Africans are sexually more performant, because it’s part of our nutrition. And once it’s part of the nutrition, it becomes a genetic matter.” – Man, 41, first generation in Belgium

Some men used stimulating plants due to perceived pressure to maintain strong sexual potency. This concept of sexual potency extended beyond the absence of erectile dysfunction and included factors like high sexual endurance and satisfying a female partner. Several male participants emphasized the benefits of these plants in delaying ejaculation, enabling prolonged sexual activity.

Seven participants noted that men face pressure to meet their sexual partners' expectations, which have become increasingly demanding. Two men expressed the need to excel as sexual partners, while one participant emphasized that a man's sexual performance significantly influences his self-esteem. Sexual potency was viewed as a fundamental aspect of being a “real” man, and three participants believed that men must be strong sexual partners to retain a woman's interest, as unsatisfactory sexual experiences could lead to relationship issues or infidelity.

“Nowadays young people want to prove themselves. I’m talking about a mental factor, a societal phenomenon as well. This encompasses weakness in sexual intercourse as well.” – Woman, 53, first generation in Belgium

“You are supposed to be the best. You're supposed to be something no one has seen before. […] She's supposed to think ‘my husband is superman.’ So, if you would use it and it doesn’t work, your girlfriend is going to say to herself, ‘actually, today he's bad’. For a man, it is impossible to be bad.”—Man, 28, second generation in Belgium

One participant described how when a man is unable to sexually satisfy a woman, he risks public humiliation. Here, sexual potency-boosting plants were actively described as a tool to combat and avoid shame about not meeting sexual expectations.

“For us, it is out of the question not to satisfy a woman. If you fail to satisfy, you will be mocked. That means that the girl can go around saying that you weren’t performing well. And that's a total shame. And so, to avoid that, I would say it is necessary to dope beforehand. That is to say take plants that can improve your performance.” – Man, 37, first generation in Belgium

#### Theme 3: taboos and social norms surrounding female sexuality

Several participants suggested that the lower availability of plants for female sexual health is influenced by taboos surrounding female sexuality. Ten participants mentioned that women tend to discuss sexuality less openly than men within their community. Eleven participants believed that women are more easily judged when expressing sexual desire. Four participants mentioned that openly expressing sexual needs could lead to assumptions of promiscuity. Additionally, four participants noted differences in social norms regarding sexuality between men and women of Congolese descent.

Two participants emphasized how a woman's value as a potential partner is significantly influenced by her sexual behavior, with multiple previous partners seen as devaluing her, making men less inclined to pursue a relationship. Two participants also noted that these social norms have caused women to fear judgment and refrain from openly discussing their sexual desires.

“Women talk about it less. They may be ashamed to talk about it. Women are embarrassed, men are not embarrassed. “ – Woman, 59, first generation in Belgium

“Women are not allowed to seek that pleasure, those things themselves. That has to come from men. If a woman uses these things (rd. plants), people are going to think 'she's in prostitution' or 'she always wants it.' With women it’s a little secret. They use that, I don't know what products, but it's a secret. But for men, yes, men can talk openly about that.” – Man, 66, first generation in Belgium

“When a woman talks to me about sexuality, I tell myself ‘that one is useless’, which is to say that she’s too experienced. She has been with a lot of men.” – Man, 37, first generation in Belgium

Women’s reluctance to communicate openly about sex seemed not limited to public interactions but also manifested itself in participants' private sphere. Four (mostly female) participants described how women risk the same judgment when discussing their sexual desires or dissatisfaction with their sexual partner. Five participants actively reported a lack of communication between sexual and or romantic partners.

“There is a problem of communication. You can’t tell your husband that you’re not satisfied in bed. He will accuse you of being a slut. So even when you’re not happy, you accept it. You accept it and make children. Right now, they say that women cheat… but it’s because there is no communication.” – Woman, 59, first generation in Belgium

Despite the dominant narrative of taboos and judgments surrounding female sexuality, five participants also highlighted that society is evolving and moving away from unequal gender norms:

“We are no longer at the time when the woman is not being satisfied, that she had to be beautiful and shut up” – woman, 53, first generation in Belgium

## Discussion

In this exploratory study, we documented medicinal plant use for sexual health within the Congolese community in Belgium. While we aimed to identify commonly used plant species in the context of sexual health, our list may not encompass all plants and plant products used in this context among people of Congolese descent in Belgium. We examined perceptions of sexuality and gender norms related to medicinal plant use. It is important to note that these findings are limited to this context and cannot be seen apart from this type of plant use. Methodological limitations include a larger number of male participants, primarily due to challenges in recruiting young women who were willing to discuss these topics. Our research primarily represents heteronormative perspectives, as all participants identified as heterosexual and cisgender. The researcher's outsider status and colonial history may have initially led to mistrust but also facilitated open conversations [43]. However, discussing intimate topics with a female interviewer may have influenced male participants' willingness to discuss sexual potency.

### Knowledge of Congolese medicinal plants in Belgium

Medicinal plants targeting sexual health were widely used and available in Belgium. Both the extent of plant use and motivations behind consumption strongly differed between men and women. The majority of reported plant species were primarily used to enhance male sexual potency. This finding aligns with a previous market survey of African medicinal plants conducted in Matongé, which identified male aphrodisiacs as one of the most prominent use categories [24]. Results suggest that for men, plants used for sexual health serve a dual purpose. Firstly, they are considered remedies for potency issues such as erectile dysfunction and premature ejaculation. Secondly, men use these plants as sexual performance enhancers, meaning that men who do not have any specific sexual potency problems consume these plants in order to boost their overall sexual prowess. This dual use is consistent with reports of high incidences of recreational use of aphrodisiacs in DR Congo and other Sub-Saharan African countries, as well as the Caribbean [44–48]. A study conducted among teenaged to middle-aged men in Kinshasa revealed that over half of the respondents had experience using libido- or erection-enhancing products. Notably, 75% of aphrodisiac consumers justified their use based on the pursuit of enhanced sexual performance in the form of prolonged intercourse, while only 19.4% cited erectile dysfunction as their primary reason for consumption [46].

For women, plant uses and applications were different compared to those for men. Only a third of the mentioned herbal products had reported applications in female sexual and reproductive health, primarily for treating vaginal infections, indicating a focus on ailment-centered usage. Limited knowledge was documented regarding plant species with potential applications in female sexual health. The presence of an informant bias, evident in the smaller number of interviewed women, may have led to an underestimation of female applications. However, most female participants believed herbal medicines were uncommonly used by women for sexual health. In contrast, van Andel and Fundiko [24] reported “women's health” as the largest category of African medicinal plant applications in Matongé. Differences in research scope and participant groups may explain this disparity. While Van Andel and Fundiko [24] documented herbal medicine use across various applications, our study specifically focused on sexual health. Women's sexual health encompasses ailments beyond our study's scope, such as menstrual pain and postpartum hemorrhages, likely contributing to a broader range of herbal practices.

Most plant species with male sexual potency-enhancing properties had similar documented uses in studies conducted in DR Congo [47–49]. The frequently mentioned herbal powder *ankoro* was absent from consulted ethnobotanical literature, despite being recognized as a popular aphrodisiac in Kinshasa and marketed online as a Congolese aphrodisiac [46]. The unidentified plant species known as mogomboro is mentioned several times in ethnobotanical literature but is associated with multiple plant species (*Acridocarpus* sp. [21] and *Buchholzia macrophylla* Pax [47]). Besides, *mogomboro* appeared in several online news articles reporting the commercialization of aphrodisiacs in Kinshasa [33, 34]. Due to the vernacular names of both plant products being linked with various plant species in the scientific literature, identification through existing literature is not feasible. This also underscores the incomplete state of ethnobotanical research in the DR Congo. Unprescribed Viagra and the drink ‘Asili Power Plus – haute énergie’, mentioned by male participants, ranked among the top three most commonly used aphrodisiacs in a Kinshasa study, which might result from a preference for commercialized, man-made products in that context, potentially devaluing herbal alternatives [46]. The use of *C. sativus* and *T. cacao* for libido-stimulating purposes is considered new applications adopted after migration. Presumably, the first is being used as a libido enhancer as a result of symbolic association, initiated by its phallic shape.

Plants with potential applications in addressing female sexual and reproductive health, though frequently documented in Congolese ethnobotanical literature, rarely included female sexual health practices. However, exceptions were found in studies specifically focusing on female sexual health herbal practices [50–52]. For instance, a study in Kinshasa addressed women's use of plants for intimate hygiene, including three of the five plant species identified in our study for combatting vaginal infections (*M. indica, Z. officinale,* and *Ocimum gratissimum* L.). These plants were used for various purposes, including reducing vaginal lubrication and narrowing the vaginal canal, practices common in several (including African) cultures and reported to be continued among people of Surinamese descent in the Netherlands [5]. Interestingly, these practices were not mentioned in Belgium, possibly due to the loss of practices or their association with taboos.

Our research reveals a significant gender bias in the use of medicinal plants for sexual health. Men reportedly face substantial social pressure to conform to cultural expectations of male sexuality, consistent with previous studies [53, 54]. Participants highlighted sexual potency as a crucial aspect of masculinity, defined by a man's ability to satisfy a woman sexually, including female orgasms and lasting intercourse. Failing to meet these expectations could undermine a man's self-perception as a "real" man. Some men using sexually stimulating plants aimed to match women's sexual stamina, seeking to impress them and viewing women as judges of masculinity. Farvid and Braun [55] noted that sexual performance plays a central role in a man's ego and sense of masculinity, giving women a degree of “power” over men by highlighting sexual shortcomings. Our findings support this idea, as a male participant described how a woman's disclosure of his inability to satisfy her could be deeply shameful. Consequently, men's masculinity is scrutinized by their environment through the execution and vocalization of sexual acts [56].

In response to societal pressure for strong sexual potency, some Congolese men in Belgium use sexually stimulating plants to enhance their sexual prowess and meet embedded masculinity norms related to sexual performance. This motivation aligns with observations of using both herbal and non-herbal sexual enhancers to improve performance and meet sexual expectations in several Sub-Saharan African countries [44, 46]. In Kinshasa, the popularity of aphrodisiacs among young men has sparked media debates. Young men view sex as a competition, striving to impress their female partners with exceptional performances, leading to widespread plant consumption as sexual enhancers [33, 57]. These Kinshasa observations mirror our findings in Brussels.

Some male participants linked sexual potency to Africanness or blackness [56], believing that individuals of African descent are inherently more sexually performant and focused on sexuality compared to those from other backgrounds. They attributed this belief to the longstanding Congolese tradition of consuming sexually stimulating plants, suggesting that Congolese bodies are prepared from a young age for heightened sexual potency. However, it is crucial to acknowledge that this self-presentation may be influenced by the historical hypersexualization of people of African descent in Western contexts. Persistent racial stereotypes depict black African men as sexually promiscuous, hypermasculine, and dominant, rooted in the historical dehumanization of black people during colonial times [56]. These stereotypes persist through various media forms and continue to shape perceptions of black men's sexuality [58]. Our findings suggest that narratives of black hypersexuality may have influenced the self-perceptions of black men, imposing additional pressure in terms of sexual expectations. In a Western context, black men must not only conform to prevailing local ideals of male sexuality but also contend with perceptions of African hypersexuality.

### Taboos and social norms surrounding female sexuality

The lower availability and knowledge of plants targeting female sexual health are attributed to taboos surrounding female sexuality. Congolese women are generally less open about sexuality compared to men, potentially reducing demand for plants targeting female sexual health. Engaging in the trade of these plants may imply a desire for sexual enhancement or the presence of sexual ailments, which culturally may be more challenging for women. Negative connotations surrounding herbal products used for female sexual health were also noted in Kinshasa by Kabena et al. [52]. Despite interviews leading to a list of 37 plant species used for intimate female hygiene, only about eight percent of the interviewed women claimed to use them personally. The latter researchers attributed this discrepancy to the cosmopolitan setting, where plant-based practices for female intimate hygiene may be perceived as ‘degrading and shameful’. This raises an important question regarding the devaluation of plant remedies in urban societies: has the devaluation resulted in a decrease of the use of these herbal remedies or has usage continued despite the establishment of a taboo around their use, thereby hindering participants from openly discussing their personal use of them? Intriguingly, while female plant use for sexual health was described as shameful, the trade in men-focused aphrodisiacs thrives in Kinshasa, evidencing a contrasting perception of plant use for sexual health between genders.

Our study reveals gendered norms regarding socially acceptable sexual behavior. Women face stricter judgments based on their sexual behavior, with having multiple previous sexual partners devaluing them and reducing their chances of being considered potential partners by men. This aligns with the sexual double standard (SDS) concept, where similar sexual behaviors are differentially appreciated based on the actor's gender [59]. Our findings illustrate an SDS within the Congolese community in Belgium, where women are judged more harshly for openly discussing their sexual desires and experiences compared to men, who are more readily accepted and even praised for such actions. This judgment has created a taboo around female sexuality, making women reluctant to discuss sexual experiences and expectations, both openly and with their male partners. However, participants also mentioned an ongoing shift toward more gender-equitable norms within the Congolese community in Belgium, diverging from traditional Congolese notions.

Multiple studies have highlighted prevalent gender inequitable norms in DR Congo [60–62]. These gender dynamics have been significantly influenced by Western ideologies throughout history and continue to be impacted by them. In precolonial times, women's status and access to resources varied across cultural and political contexts. However, the colonial period imposed Christian-European gender roles, making men the primary breadwinners and heads of households, and women were expected to be docile housewives within heterosexual nuclear families. These concepts have persisted through churches and continue to shape discussions on gender norms in DR Congo and other parts of Africa today [63–65].

### Implications and future perspectives

The use of Congolese medicinal plants for sexual health applications carries significant implications regarding health, healthcare, and biodiversity conservation, as well as social implications. Firstly, the consumption of herbal products can lead to serious negative side effects alongside the intended treatment. Herbal products often lack clear information regarding their posology. Consequently, users often resort to trial and error, which may result in overconsumption with harmful health consequences. Moreover, when combined with allopathic drugs, herbal product–drug interactions and potential adverse effects on other existing medical conditions may occur [66]. This emphasizes the importance of informing Western healthcare workers working with migrant communities about common herbal practices.

Additionally, unsustainable harvest of natural resources and their uncontrolled commercial export and import might pose threats to biodiversity. *Mondia whitei,* for instance, was widely known among participants and can be accessed in Matongé, despite its endangered status [67]. Similarly, *Garcinia kola* is a popular medicinal plant in West and Central Africa as well as in Matongé, but its wild populations are declining due to overexploitation [68]. However, for the majority of plant species, the exact impacts of commercial extraction are still unclear. Factors contributing to this are limited information on plant distributions and large aspects of the trade occurring through informal networks [69].

On another level, the use of plants as sexual stimulators may have social implications. Relying on herbal stimulants may give users a sense of dependency, which could further amplify feelings of inadequacy and anxiety [70].

This underscores the need for further research that focuses on assessing species vulnerability and the impacts of (semi-)commercial extraction on commonly used medicinal plant species, as well as the identification of potential side effects of medicinal plants and herb–drug interactions, and the broader social consequences that may arise from the use of sexually stimulating plants.

## Conclusions

This paper offers insights into the use of plants for sexual health purposes among individuals of Congolese origin in Belgium, along with the underlying perceptions driving their use. The study reveals that medicinal plants targeting sexual health are widely used and available. Seventeen plant species used for sexual health purposes were inventoried, of which 14 were successfully identified and classified into 12 families. We documented a clear gender bias in plant use: plants enhancing male sexual potency, serving both as remedies for potency issues as well as sexual enhancers, were widely known and consumed. In contrast, female applications were limited and predominantly ailment-based. Most plant species’ medicinal uses were confirmed by Congolese ethnobotanical literature and included similar sexual health applications. Our results indicate that men tend to face significant social pressure to conform to cultural expectations of male sexuality. Our findings also reveal a sexual double standard within the Congolese community, as female sexuality is taboo and women face harsh judgments for openly communicating about sexual desires and experiences, while men are more readily accepted and even praised for these very actions. The use of medicinal plants for sexual health purposes may have adverse effects on users' health, significant social implications, and raise concerns regarding the conservation of plant species, which underscores the need for further research focusing on these factors. As the present study serves as an initial exploration, more in-depth future research is needed to fully unravel the use of medicinal plants for sexual purposes, particularly to understand the role of Congolese herbal practices for female sexual health in Belgium.

## Data Availability

The datasets used and/or analyzed during the current study are available from the corresponding author on reasonable request.

## Abbreviations

DR Congo: Democratic Republic of Congo
SDS: Sexual double standard

## References

[1] Šantić Z, Pravdić N, Bevanda M, Galić K (2017). The historical use of medicinal plants in traditional and scientific medicine. Psychiatr Danub.

[2] Stanifer JW, Kilonzo K, Wang D, Su G, Mao W, Zhang L, Zhang L, Nayak-Rao S, Miranda JJ (2017). Traditional medicines and kidney disease in low- and middle-income countries: opportunities and challenges. Semin Nephrol.

[3] Kibungu P, Nzuki Bakwaye F, Belesi Katula H, Vanhove W, Van Damme P (2021). Ethnobotanical characterization of medicinal plants used in Kisantu and Mbanza-Ngungu territories, Kongo-Central Province in DR Congo. J Ethnobiol Ethnomed.

[4] Osamor PE, Owumi BE (2010). Complementary and alternative medicine in the management of hypertension in an urban Nigerian community. BMC Complem Alter Med.

[5] Van Andel T, Westers P (2012). Why Surinamese migrants in the Netherlands continue to use medicinal herbs from their home country. J Ethnopharmacol.

[6] Van Andel T, Carvalheiro LG (2013). Why urban citizens in developing countries use traditional medicines: the case of suriname. Evid Complem Alter Med.

[7] Ekor M (2014). The growing use of herbal medicines: issues relating to adverse reactions and challenges in monitoring safety. Front Pharmacol.

[8] Ernst E, White A (2000). The BBC survey of complementary medicine use in the UK. Complem Ther Med.

[9] Tindle H, Davis R, Eisenberg D, Phillips R (2005). Trends in use of complementary and alternative medicine by us adults: 1997–2002. Alter Therap Health Med.

[10] Ahmad KMS, Ahmad I (2019). Herbal medicine: current trends and future prospects. New Look Phytomed Adv Herbal Products Novel Drug Leads.

[11] Timoshyna A, Ke Z, Yang Y, Ling X. Controlling the invisible trade: wild plant resources and their sustainability. WCO News Magazine. 2020.

[12] Balick MJ, Kronenberg F, Ososki AL, Reiff M, Fugh-berman A, O’Connor B, Roble M, Lohr P, Atha D (2000). Medicinal plants used by latino healers for women’s health conditions in New York city 1. Econ Bot.

[13] Medeiros PM, De Soldati GT, Alencar NL, Vandebroek I, Pieroni A, Hanazaki N, De Albuquerque UP (2012). The use of medicinal plants by migrant people: adaptation, maintenance, and replacement. Evid Complem Alter Med.

[14] Ceuterick M, Vandebroek I (2017). Identity in a medicine cabinet: Discursive positions of Andean migrants towards their use of herbal remedies in the United Kingdom. Soc Sci Med.

[15] Moullan Y, Jusot F (2014). Why is the “healthy immigrant effect” different between European countries?. Eur J Pub Health.

[16] Rosano A, Dauvrin M, Buttiġieġ SC, Ronda E, Tafforeau J, Dias S (2017). Migrant’s access to preventive health services in five EU countries. BMC Health Serv Res.

[17] Sasse A, Deblonde J, De Rouck M, Montourcy M, Van Beckhoven D. Epidemiologie van AIDS en HIV-Infectie in België. n.d. https://www.sciensano.be/sites/default/files/report_sida_2019_nl_final_february2020_1.pdf. Accessed 1 Feb 2024.

[18] Schoonvaere Q. Studie over de Congolese migratie en de impact ervan op de Congolese aanwezigheid in België: Analyse van de voornaamste demografische gegevens. 2010.

[19] Van Reybrouck D. Congo, een geschiedenis. De Bezige Bij, 2010.

[20] Demart S (2013). Congolese migration to Belgium and postcolonial perspectives. Afr Diaspora.

[21] Beddington E. Out of Africa: Brussels’ vibrant Matonge quarter. The Guardian. 2013. https://www.theguardian.com/travel/2013/nov/03/brussels-matonge-quarter-belgium. Accessed 16 June 2023.

[22] Demart S. Histoire orale à Matonge (Bruxelles): un miroir postcolonial. Revue Européenne Des Migrations Internationales, 2013;29. 10.4000/remi.6323

[23] Gombeer S, Nebesse C, Musaba P, Ngoy S, Peeters M, Vanderheyden A, Meganck K, Smitz N, Geers F, Van Den Heuvel S, Backeljau T, De Meyer M, Verheyen E (2021). Exploring the bushmeat market in Brussels, Belgium: a clandestine luxury business. Biodivers Conserv.

[24] Van Andel T, Fundiiko MCC (2016). The trade in african medicinal plants in matonge-ixelles Brussels (Belgium). Econ Botany.

[25] Vawazola NVH, Tshiendesha KC, Lumfwankenda MLS, Ntumba D, Kilemba N, Binga N, Masimango NJ-T. Inventaire des produits végétaux entrant dans la préparation des boissons Tangawisi. Congo Sciences. 2018. 6(3).

[26] De Meyer E, Van Damme P, de la Peña E, Ceuterick M (2022). ‘A disease like any other’ traditional, complementary and alternative medicine use and perspectives in the context of COVID-19 among the Congolese community in Belgium. J Ethnobiol Ethnomed.

[27] Yaga Burundi. Plantes traditionnelles comme stimulant sexuel : prudence. 2013. https://www.yaga-burundi.com/urukundo/plantes-traditionnelles-comme-stimulant-sexuel-prudence/. Accessed 16 June 2023.

[28] Seleteng Kose L, Moteetee A, Van Vuuren S (2015). Ethnobotanical survey of medicinal plants used in the Maseru district of Lesotho. J Ethnopharmacol.

[29] Towns AM, Quiroz D, Guinee L, De Boer H, Van Andel T (2014). Volume, value and floristic diversity of Gabons medicinal plant markets. J Ethnopharmacol.

[30] Chiribagula VB, Alonie-Gracia A, Mahungala AK, Amuri SB, Ndjolo PO (2020). Ethnobotanical study of medicinal plants used in the treatment of sexual dysfunctions in traditional medicine in Kampemba-Lubumbashi, DR Congo. World J Adv Res Rev.

[31] Nzuki Bakwayé F, Termote C, Kibungu P, Van Damme P. Identification et importance locale des plantes médicinales utilisées dans la région de Mbanza-Ngungu, République démocratique du Congo. Bois et Forêts Des Tropiques. 2013;316.

[32] M’Buy SH. Mode et mœurs : des kinois de plus en plus dépendants des aphrodisiaques. 2016. http://creationactive.blogspot.com/2016/12/mode-et-murs-des-kinois-de-plus-en-plus.html. Accessed 16 June 2023.

[33] Ntumba M. Sexualité : la prise des aphrodisiaques s’enracine dans les habitudes des jeunes kinois. Lemag. 2023. https://www.lemag.cd/sante/2023/04/11/sexualite-la-prise-des-aphrodisiaques-senracine-dans-les-habitudes-des-jeunes. Accessed 16 June 2023.

[34] RedactionSA. RDC : comment se porte le commerce des aphrodisiaques à Kinshasa ? Sahuti Africa. 2022. https://sahutiafrica.net/rdc-comment-le-commerce-des-aphrodisiaques-marche-a-kinshasa/. Accessed 16 June 2023.

[35] Robins SG, Lee HC, Lim RSH, Fullerton J (2010). Recruitment of hard-to-reach population subgroups via adaptations of the snowball sampling strategy. Nurs Health Sci.

[36] Dolores M, Tongco C (2007). Purposive sampling as a tool for informant selection. Ethnobot Res Appl.

[37] Saunders B, Sim J, Kingstone T, Baker S, Waterfield J, Bartlam B, Burroughs H, Jinks C (2018). Saturation in qualitative research: exploring its conceptualization and operationalization. Qual Quant.

[38] Braun V, Clarke V (2006). Using thematic analysis in psychology. Qual Res Psychol.

[39] PROTA4U. Plant Resources of the World (PROW). 2023. https://prota.prota4u.org/. Accessed 16 June 2023.

[40] Plantentuin Meise. The Flora of Central Africa. 2023. https://www.floredafriquecentrale.be/#/en/home. Accessed 16 June 2023.

[41] IPNI. International Plant Names Index. Published on the Internet http://www.ipni.org, The Royal Botanic Gardens, Kew, Harvard University Herbaria and Libraries and Australian National Herbarium. 2023. https://www.ipni.org/. Accessed 15 June 2023.

[42] Bhat R, Karim AA (2010). Tongkat Ali (*Eurycoma longifolia* Jack): a review on its ethnobotany and pharmacological importance. Fitoterapia.

[43] Darwin Holmes AG (2020). Researcher positionality: a consideration of its influence and place in qualitative research—a new researcher guide. Shanlax Int J Edu.

[44] Adamu H, Adamu AA, Cletus B, Marafa L, Abdulmalik M (2022). Prevalence and pattern of aphrodisiac use among adult population in Sokoto Metropolis, Northwest Nigeria. Int J Res Rep Gynaecol.

[45] Manortey S, Mensah PA, Acheampong GK (2018). Evaluating factors associated with the use of aphrodisiacs among adult male residents in Ashaiman municipality. Ghana OALib.

[46] Ndombe DM, Manun’Ebo MF, Ilunga BM (2022). Consommation Des Aphrodisiaques Chez Les adolescents Et Adultes À Kinshasa: Prévalence Et Facteurs Associés. Eur Sci J ESJ.

[47] Van Andel T, Mitchell S, Volpato G, Vandebroek I, Swier J, Ruysschaert S, Jiménez CAR, Raes N (2012). In search of the perfect aphrodisiac: parallel use of bitter tonics in West Africa and the Caribbean. J Ethnopharmacol.

[48] Fiaveh DY (2020). Masculinity, male sexual virility, and use of aphrodisiacs in Ghana. J Men’s Stud.

[49] Kibungu Kembelo AO. Quelques plantes médicinales du Bas-Congo et leurs usages. n.d. http://www.ethnopharmacologia.org/prelude2020/pdf/biblio-hk-61-kibungu.pdf. Accessed 16 June 2023.

[50] Mola AM, Idrissa AZ, Mwange RK, Mukeba FB, Cakupewa MF, Mangambu JDD, Balagizi IK (2022). Ethnobotanical survey of plants used against erectile dysfunction in the commune of Ngaba in Kinshasa/DR Congo. World J Adv Res Rev.

[51] Ngbolua KN, Inkoto CL, Mongo NL, Ashande CM, Masens YB, Mpiana PT (2019). Étude ethnobotanique et floristique de quelques plantes médicinales commercialisées à Kinshasa, République Démocratique du Congo. Revue Marocaine Des Sciences Agronomiques et Vétérinaires.

[52] Ali-Risasi C, Verdonck K, Padalko E, Vanden Broeck D, Praet M (2015). Prevalence and risk factors for cancer of the uterine cervix among women living in Kinshasa, the Democratic Republic of the Congo: a cross-sectional study. Infect Agents Cancer.

[53] Kabena NO, Lukoki LF, Mpiana TP, Ngombe KN, Ruphin PF, Robijaona B, Koto-te-Nyiwa N (2014). Phytochemical screening of some medicinal plants traditionally used by African Women in Kinshasa city (DR Congo) for their intimate hygiene and evaluation of the pH of derived recipes. J Mod Drug Discov Drug Deliv Res.

[54] Kabena N, Ngombe K, Ngbolua K, Kikufi B, Lassa L, Mboloko E, Mpiana P, Lukoki L (2015). Etudes ethnobotanique et écologique des plantes d’hygiène intime féminine utilisées à Kinshasa (République Démocratique du Congo). Int J Biol Chem Sci.

[55] Huysamen M (2020). “There’s massive pressure to please her”: on the discursive production of men’s desire to pay for sex. J Sex Res.

[56] Murray SH (2018). Heterosexual men’s sexual desire: supported by, or deviating from, traditional masculinity norms and sexual scripts?. Sex Roles.

[57] Farvid P, Braun V (2006). “Most of us guys are raring to go anytime, anyplace, anywhere”: male and female sexuality in Cleo and Cosmo. In Sex Roles.

[58] Spronk R (2014). The idea of African men: dealing with the cultural contradictions of sex in academia and in Kenya. Cult Health Sex.

[59] Momath D. Les jeunes accros aux aphrodisiaques. Habari RDC. 2016. https://habarirdc.net/jeunes-accros-aux-aphrodisiaques/. Accessed 17 June 2023.

[60] Wilkins AC (2012). STIGMA AND STATUS: interracial intimacy and intersectional identities among black college men. Gend Soc.

[61] Gómez-Berrocal C, Moyano N, Álvarez-Muelas A, Sierra JC (2022). Sexual double standard: a gender-based prejudice referring to sexual freedom and sexual shyness. Front Psychol.

[62] Cislaghi B, Bhatia A, Li M, Lian Q, Baird S, Kayembe P, Chipeta E, Moreau C (2021). Changes in the sexual double standard associated with sociodevelopmental factors among young adolescents in Kinshasa. J Adolesc Health.

[63] Lusey H, San Sebastian M, Christianson M, Edin KE (2018). Prevalence and correlates of gender inequitable norms among young, church-going women and men in Kinshasa, Democratic Republic of Congo. BMC Public Health.

[64] Slegh H, Barker G, Levtov R. Gender Relations, Sexual and Gender-Based Violence and the Effects of Conflict on Women and Men in North Kivu, Eastern Democratic Republic of the Congo: Results from the International Men and Gender Equality Survey. Final Report. 2014. IMAGES. https://promundo.org.br/wp-content/uploads/2014/12/Gender-Relations-Sexual-and-Gender-Based-Violence-and-the-Effects-of-Conflict-on-Women-and-Men-in-North-Kivu-Eastern-DRC-Results-from-IMAGES.pdf. Accessed 1 Dec 2023.

[65] Lauro A. Women in the Democratic Republic of Congo. In Oxford Research Encyclopedia of African History. Oxford University Press. 2020. 10.1093/acrefore/9780190277734.013.544

[66] Mertens C, Myrttinen H (2019). ‘A real woman waits’–heteronormative respectability, neo-liberal betterment and echoes of coloniality in SGBV programming in eastern DR Congo. J Interv Statebuild.

[67] Tamale S (2014). Exploring the contours of African sexualities: religion, law and power. Afr Hum Rights Law J.

[68] Fugh-Berman A (2000). Herb-drug interactions. Lancet.

[69] Aremu AO, Cheesman L, Finnie JF, Van Staden J (2011). *Mondia whitei* (Apocynaceae): a review of its biological activities, conservation strategies and economic potential. S Afr J Bot.

[70] Maňourová A, Chinheya IP, Kalousová M, Ruiz-Chután JA, Okafor UC, Tchoundjeu Z, Tsobeng A, Van Damme P, Lojka B (2023). Domestication Potential of *Garcinia kola* Heckel (Clusiaceae): searching for diversity in South Cameroon. Plants.

[71] Van Andel T, Croft S, van Loon EE, Quiroz D, Towns AM, Raes N (2015). Prioritizing West African medicinal plants for conservation and sustainable extraction studies based on market surveys and species distribution models. Biol Cons.

[72] Both R (2016). A matter of sexual confidence: young men’s non-prescription use of Viagra in Addis Ababa, Ethiopia. Cult Health Sex.

[73] American Anthropological Association (AAA). Code of Ethics of the American Anthropological Association. 2012. http://ethics.americananthro.org/. Accessed 16 June 2023.

[74] ISE/International Society of Ethnobiology. 2006. Code of Ethics. https://www.ethnobiology.net/what-we-do/core-programs/ise-ethics-program/code-of-ethics/ Accessed 16 June 2023.

[75] Tchicaillat-Landou M, Petit J, Gaiani C, Miabangana ES, Kimbonguila A, Nzikou JM, Scher J, Matos L (2018). Ethnobotanical study of medicinal plants used by traditional healers for the treatment of oxidative stress-related diseases in the Congo Basin. J Herbal Med.

[76] Katemo M, Mpiana PT, Mbala BM, Mihigo SO, Ngbolua KN, Tshibangu DST, Koyange PR (2012). Ethnopharmacological survey of plants used against diabetes in Kisangani city (DR Congo). J Ethnopharmacol.

[77] Termote C, van Damme P, Djailo BD (2010). Eating from the wild: Turumbu Indigenous knowledge on noncultivated edible plants, Tshopo district, DR Congo. Ecol Food Nutr.

[78] Mbuta KK, Mwima K, Bitengeli M, Y’okolo I, Kavuna M, Mandanga M, Kalambayi M, Izamajole N, Kazembe K, Booto K, Vasaki N, Mwabonsika B, Lody D. Plantes médicinales de traditions : Province de l’Équateur - R.D. Congo. Institut de recherche en sciences de la santé. 2012. http://www.nzenzeflowerspauwels.be/bikonda-ku-mbuta.pdf. Accessed 16 June 2023.

